# First person – Li Wang

**DOI:** 10.1242/dmm.049322

**Published:** 2021-11-11

## Abstract

First Person is a series of interviews with the first authors of a selection of papers published in Disease Models & Mechanisms, helping early-career researchers promote themselves alongside their papers. Li Wang is first author on ‘ Suppressing STAT3 activity protects the endothelial barrier from VEGF-mediated vascular permeability’, published in DMM. Li is a postdoctoral fellow in the lab of Luke Hoeppner at the University of Minnesota, Austin, MN, USA, investigating dysregulation of vascular permeability in the pathology of several human diseases using zebrafish, mouse and cultured human endothelial cells as models.


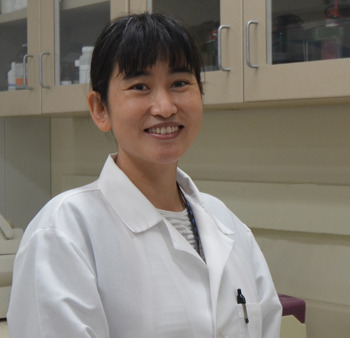


**Li Wang**

**How would you explain the main findings of your paper to non-scientific family and friends?**

Proper vascular function is essential for maintaining good health. Our body's vascular system distributes oxygenated blood to all tissues, returns deoxygenated blood to the lungs, and helps maintain balanced function among blood cells, lipids and immune cells. During tissue injury or disease, vascular barrier integrity is impaired. A protein called vascular endothelial growth factor (VEGF) is a central mediator of vascular permeability. Increased VEGF expression promotes hyperpermeability, oedema and tissue damage, which contribute to the pathogenesis of cardiovascular disease, cerebrovascular conditions, cancer, retinal disorders and acute lung injury. However, VEGF also plays a role in tissue repair following damage by stimulating the growth of new blood vessels. Our goal is to discover molecular signalling that can be targeted to reduce the tissue-damaging effects of vascular permeability, while not affecting the tissue-repair role of VEGF. In our study, we demonstrate that VEGF signals through a mediator called signal transducer and activator of transcription 3 (STAT3) to promote vascular permeability. In mice engineered for STAT3 deletion in their vascular (i.e. endothelial) cells, we observe reduced VEGF-induced vascular permeability. Similarly, in Stat3 knockout zebrafish, vascular barrier integrity is maintained despite ‘turning on’ VEGF expression. Importantly, the process of blood vessel formation stimulated by VEGF is unaffected in these animals, which suggests that the function of VEGF in tissue repair is maintained upon STAT3 inhibition. STAT3 can be effectively inhibited using a drug called pyrimethamine, which has been US Food and Drug Administration (FDA) approved to treat parasite diseases. We show that the STAT3 inhibitor pyrimethamine substantially reduces VEGF-induced vascular permeability in zebrafish, mouse and human endothelium. STAT3 could be used as a target to reduce the pathological effects of vascular permeability in human diseases, including vascular conditions, cancer, eye disorders and COVID-19-associated acute lung injury.

“Our research highlights the importance of STAT3 as a central mediator of vascular permeability.”

**What are the potential implications of these results for your field of research?**

Our research highlights the importance of STAT3 as a central mediator of vascular permeability. We demonstrate that an FDA-approved STAT3 inhibitor called pyrimethamine inhibits VEGF-mediated vascular permeability, while not affecting the tissue restorative functions of VEGF. These findings suggest that targeting STAT3 may be an effective strategy to inhibit vascular permeability, oedema, and tissue damage associated with heart disease, stroke, retinal conditions, cancer and acute lung injury.

**What are the main advantages and drawbacks of the model system you have used as it relates to the disease you are investigating?**

In our study, we took advantage of a transgenic VEGF-inducible zebrafish model that is amenable to genetic manipulation and reproducible live imaging of vascular permeability in optically clear zebrafish embryos using fluorescently labelled tracers. This model can be used to identify novel regulators of VEGF-induced vascular permeability or to test the effects of candidate drugs on vascular barrier integrity. Additionally, the widespread recent emergence of CRISPR/Cas9 genome-editing techniques has enhanced the study of genetic regulators of endothelial barrier function. As such, we crossed CRISPR/Cas9-generated Stat3 loss-of-function mutant zebrafish to the VEGF-inducible fish to study the role of STAT3 in VEGF-mediated vascular permeability. We also used endothelium tissue-specific STAT3 knockout mice, which are healthy and fertile with no overt defects in vascular development or structure. These genetic STAT3 knockout animal models could be used in other studies related to STAT3 in other diseases. One drawback of using these model systems is the time and effort required to inject tracers into the zebrafish ventricle through the pericardium and deliver Evans Blue dye into the mice by tail vein injection. However, once an individual has mastered the injection techniques, these *in vivo* models are quite useful for answering many important questions related to the molecular regulation of vascular permeability.

**Figure:**
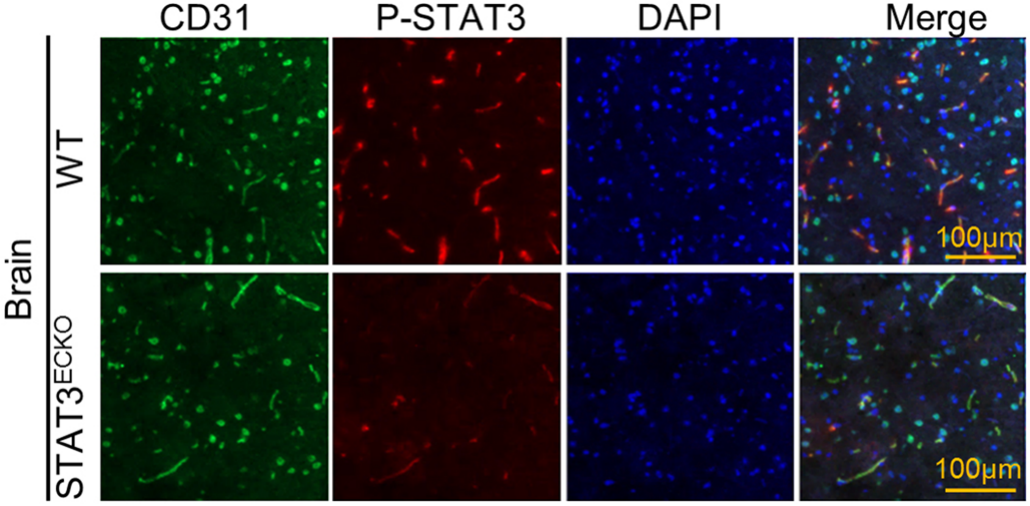
**Endothelial cell-specific STAT3 deletion in mice. **Frozen sections of brain tissue harvested from endothelial cell-specific STAT3-deficient mice (STAT3^ECKO^) or wild-type (WT) mice were immunostained for CD31 (green) and p-STAT3 (red) to identify endothelial cells and STAT3 activity, respectively. Reduced STAT3 activity was observed in endothelial cells from STAT3^ECKO^ mice relative to WT mice. Nuclei were stained blue. Images were captured using a Zeiss LSM900 confocal microscope at 20× magnification.

**What has surprised you the most while conducting your research?**

Pyrimethamine is an FDA-approved drug used to treat microbial infections. Through a chemical-biology screen, our collaborator, Dr David Frank, found that pyrimethamine is an inhibitor of STAT3 function at concentrations known to be achieved safely in humans. Dr Frank and colleagues reported that the STAT3 inhibitor pyrimethamine displays anticancer and immune-stimulatory effects in murine models of breast cancer. As highlighted in our article, we discovered that the STAT3 inhibitor pyrimethamine reduces VEGF-induced vascular permeability in mice, zebrafish and several types of human endothelial cells. I was most surprised by the power of drug repurposing – using an ‘old’ drug to treat a different disease. I am excited that pyrimethamine is already FDA approved and can be used safely in humans, which will help translate our work into the clinic.

**Describe what you think is the most significant challenge impacting your research at this time and how will this be addressed over the next 10 years?**

Discovering the molecular mechanisms underlying human disease is the first step. The next challenge is how to translate these basic science discoveries to clinical use to improve the lives of people afflicted with some of these devastating diseases. Over the next 10 years, perhaps it will become easier to synthesise drugs based on basic science findings. Pipelines to test candidate drugs in disease models will likely become more efficient and more effective. Hopefully these technical advancements will facilitate the development of new treatments and, ultimately, cures.

“[…] the advent of the preprint era has benefited early-career scientists.”

**What changes do you think could improve the professional lives of early-career scientists?**

At The Hormel Institute, University of Minnesota, many activities have been organised to improve the professional lives of early-career scientists, like external and internal seminars, career development opportunities, and the implementation of mentoring committees for postdoctoral fellows. My mentor, Dr Luke Hoeppner, also provided outstanding mentoring support throughout the whole project and has encouraged goal setting to facilitate my career growth. For most trainees, more time reading papers and more grant opportunities are desired. In regards to grant applications, I like that we can now cite preprints in proposals, and I think the advent of the preprint era has benefited early-career scientists.

**What's next for you?**

In our publication, we report that suppression of STAT3 activity inhibits vascular permeability mediated by VEGF in mice, zebrafish and human endothelium. Given that the STAT3 inhibitor pyrimethamine has already proven safe in humans, we hope to translate our findings to the clinic. To that end, we are currently testing pharmacological STAT3 inhibition in animal models of human diseases associated with vascular permeability. In terms of my own career progress, I am beginning to focus on postdoctoral grant-writing opportunities.
